# Supplementary material for: NAD+ metabolism is a key modulator of bacterial respiratory epithelial infections
Source: Nat Commun. 2023 Oct 2;14:5818. doi: 10.1038/s41467-023-41372-w (PMC10545792; doi:10.1038/s41467-023-41372-w)

Supplementary Table 1: Details on Proteome data acquisition

**reversed phase liquid chromatography (RPLC)**	
instrument	Ultimate 3000 RSLC (Thermo Fisher Scientific)
trap column	75 µm inner diameter, packed with 3 µm C18 particles (Acclaim PepMap100, Thermo Fisher Scientific)
analytical column	Accucore 150-C18, (Thermo Fisher Scientific)<br>25 cm x 75 µm, 2.6 µm C18 particles, 150 Å pore size
buffer system	binary buffer system consisting of 0.1% acetic acid, 2% ACN (buffer A) and 100% ACN in 0.1% acetic acid (buffer B)
flow rate	300 nl/min
gradient	linear gradient of buffer B from 2% up to 90%
gradient duration	0 min 2% B<br>2 min 5% B<br>10 min 5% B<br>70 min 25% B<br>75 min 40% B<br>77 min 90% B<br>82 min 90% B<br>85 min 2% B<br>95 min 2% B
column oven temperature	40°C

**mass spectrometry (MS)**	
instrument	Q Exactive mass spectrometer (Thermo Fisher Scientific)
operation mode	data-dependent
**Full MS**	
MS scan resolution	70,000
AGC target	3e6
maximum ion injection time for the MS scan	120 ms
Scan range	300 to 1650 m/z
Spectra data type	profile
**dd-MS2**	
Resolution	17,500

MS/MS AGC target	2e5
maximum ion injection time for the MS/MS scans	120 ms
Spectra data type	centroid
selection for MS/MS	10 most abundant isotope patterns with charge ≥2 from the survey scan
isolation window	3 *m/z*
Fixed first mass	100 m/z
dissociation mode	higher energy collisional dissociation (HCD)
normalized collision energy	27.5%

dynamic exclusion	30 s
Charge exclusion	1>6

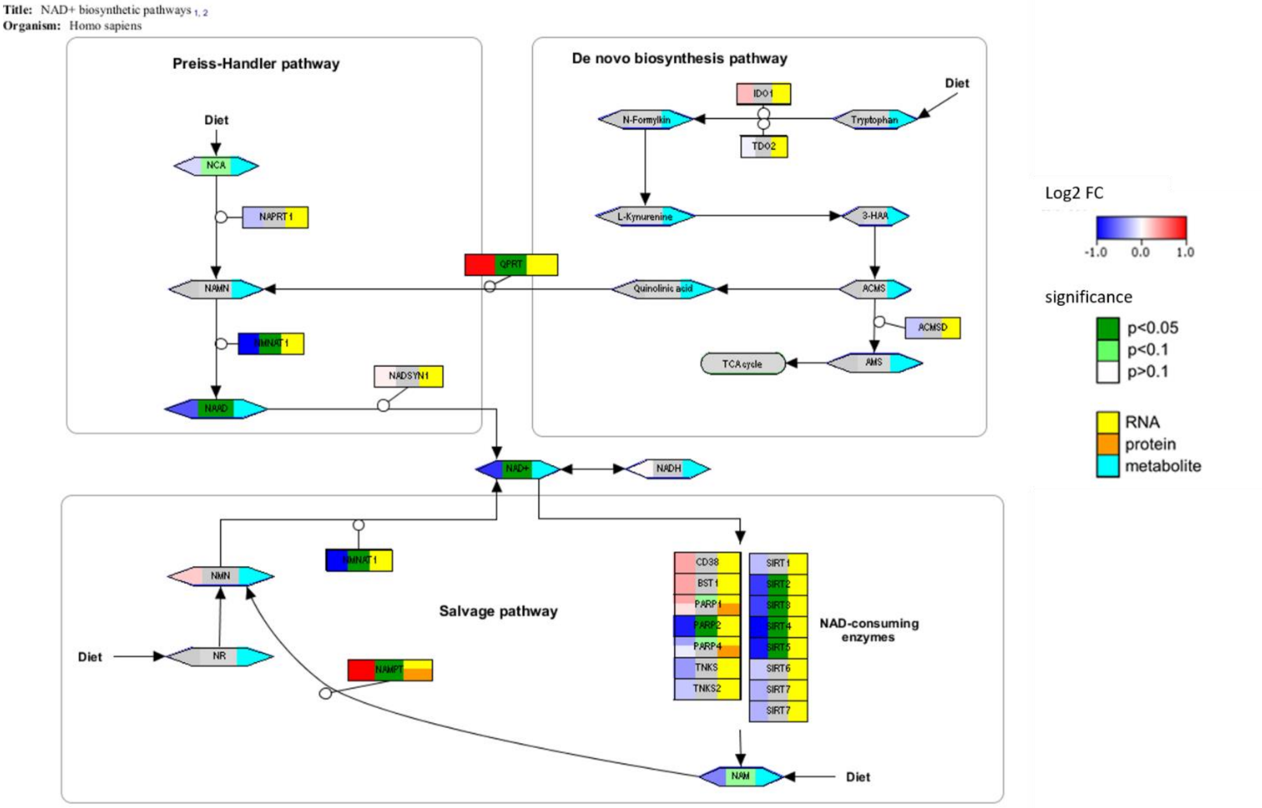

Supplementary Figure 1. Regulation of NAD^+ biosynthetic pathways upon pneumococcal infection. BEAS-2B lung epithelial cells were infected with Spn, MOI0.5 or MOI1 for 16 h. Host–cell proteome, transcriptome and intra-host–cell metabolite concentrations were measured and compared to uninfected controls. Multi-omics data was loaded into Pathvisio V3.3.0 [81] and visualized on the Wikipathways [82] Pathway WP3645 "NAD+ biosynthetic pathways" (https://www.wikipathways.org/pathways/WP3645.html).

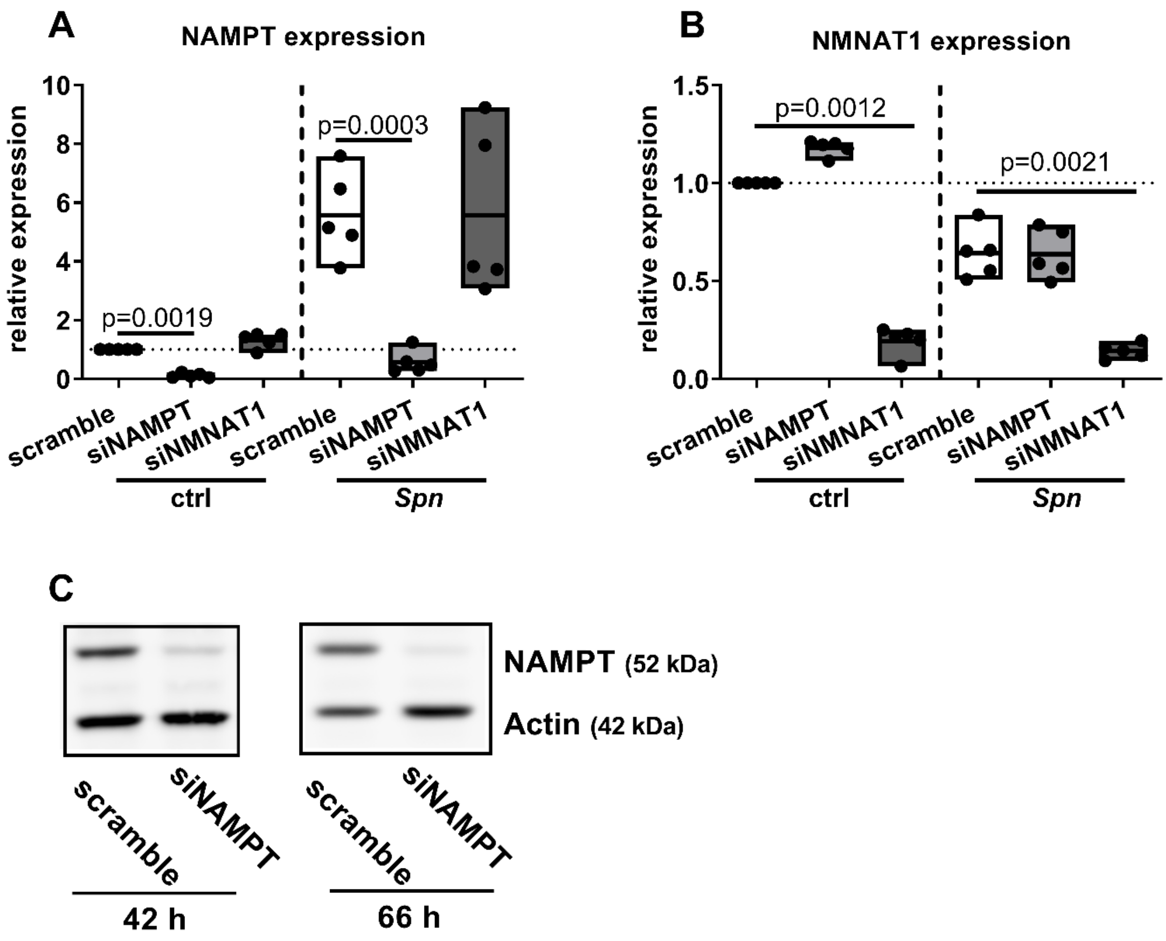

Supplementary Figure 2. siRNA transfection induced a strong knockdown of NAD$^+$ salvage genes. BEAS-2B were transfected with scramble, siNAMPT or siNMNAT1 as indicated. The medium was exchanged 48 h post transfection, and cells were either infected with Spn, MOI 1 for 16 h, or left untreated. A-B) RNA was isolated and qPCRs for indicated targets were performed. Results are normalized to the house keeping gene RPS18 and depicted relative to untreated control cells. C) Representative Western Blot analysis of NAMPT was performed after indicated time points. Statistics: two-tailed paired t test; N = 5 biologically distinct samples (A,B); 3 biologically distinct samples (C); box plots: line at mean; box ranges from min to max; results are normalized against untreated controls.

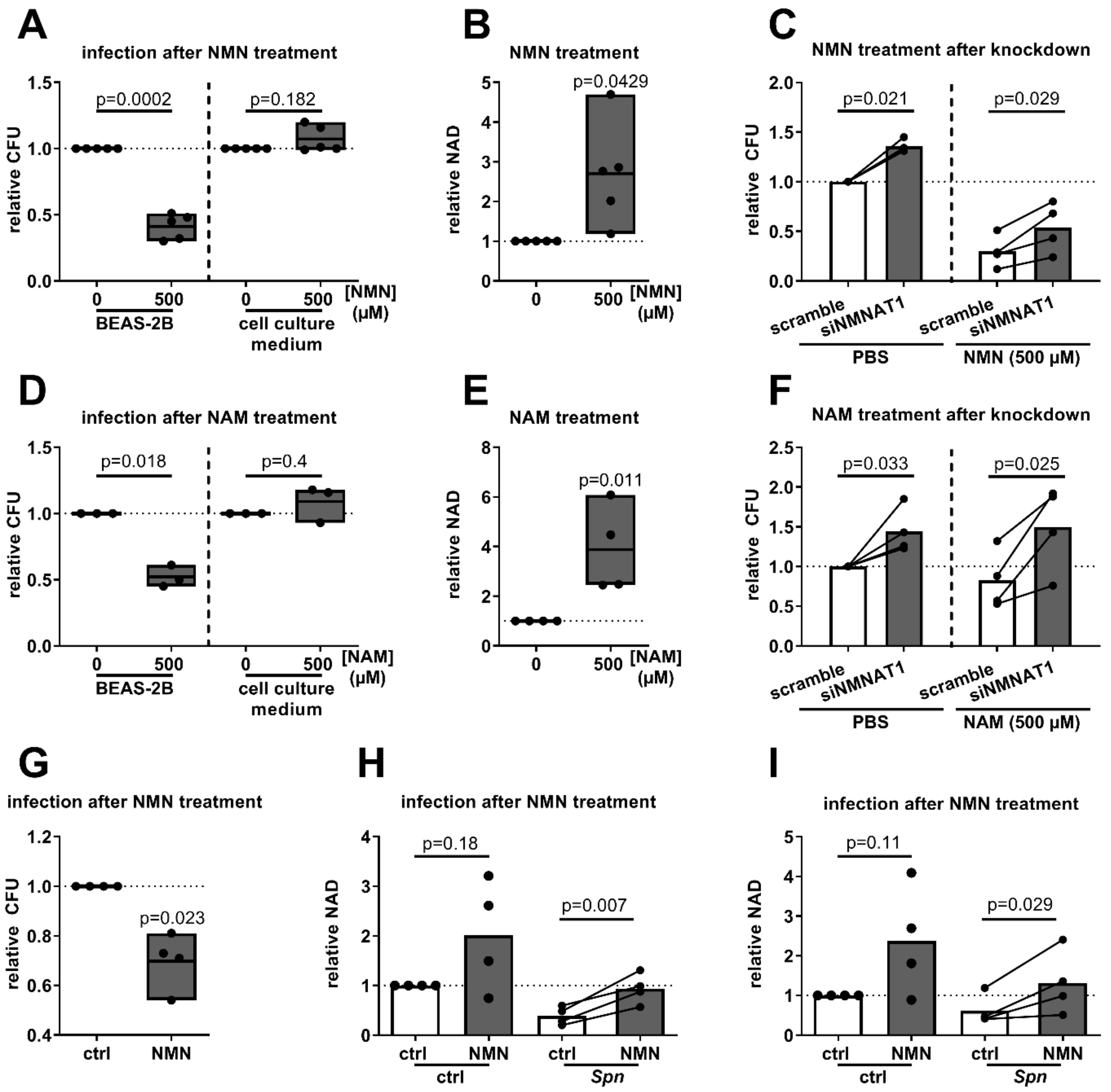

Supplementary Figure 3: NAD$^+$ precursor mediated inhibition of bacterial growth depends on host cell NAD$^+$ synthesis. A) Spn were cultured in cell-free medium or used to infect BEAS-2B at MOI 1 with and without NMN treatment. CFU were determined after 9 h and are depicted relative. B) BEAS-2B were treated with NMN for 9 h as indicated and intracellular NAD was measured. C) BEAS-2B were transfected with siNMNAT1 or a scramble control. 48 h post-transfection, cells were infected with Spn, MOI 1 or left uninfected and were treated with NMN (500 µM) or PBS. CFU/ml were determined 9 h post infection and normalized to scramble control. D) Spn were cultured in cell-free medium or used to infect BEAS-2B at MOI 1 with and without NAM treatment. CFU were determined after 9 h and are depicted relative. E) BEAS-2B were treated with NAM for 9 h as indicated and intracellular NAD was measured. F) BEAS-2B were transfected with siNMNAT1 or a scramble control. 48 h post-transfection, cells were infected with Spn, MOI 1 or left uninfected and were treated with NAM (500 µM) or PBS.

CFU/ml were determined 9 h post infection and normalized to scramble control. G-I) Primary pseudostratified human bronchial epithelial cells were cultivated at an air–liquid interface. Cells were infected apically with Spn, MOI 20 for 1 h. Afterwards, cells were washed, NMN (500 µM) was added basolateral and cells were incubated for 16 h. G, H) The amount of intracellular and extracellular NAD was determined 16 h post-infection in cell lysates (G) and apical wash fluid (H), respectively. I) Cells were washed 16 h post infection and CFU in the apical wash fluid were determined. Statistics: two-tailed paired t-test; significance was determined against uninfected/untreated controls unless indicated otherwise; N = 3 distinct samples (D); 4 biologically distinct samples (C, E-I); 5 biologically distinct samples (A, B); box plots: line at mean; box ranges from min to max; bar graphs: bar at mean; results are normalized against untreated controls.

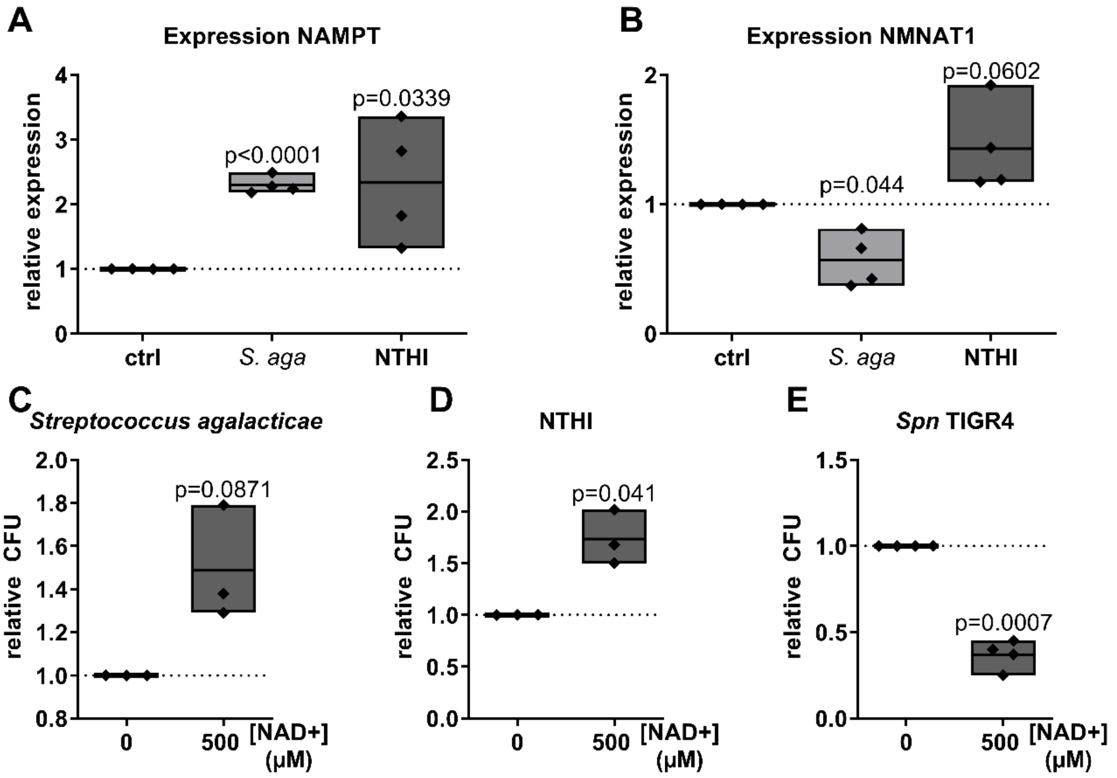

Supplementary Figure 4. NAD$^+$-mediated inhibition of bacterial replication and bacteria-induced regulation of NAD$^+$ salvage genes are not general mechanisms. A-B) BEAS-2B were infected with NTHi or S.aga, MOI1, for 16 h or left untreated for control. Afterwards, RNA was isolated and expression of indicated NAD$^+$ metabolic genes analyzed by qPCR. C-E) S.aga, NTHi and Spn TIGR4 were cultivated in BEGM for 6 h (TIGR4, S.aga) or 4 h (NTHi) and treated with 500 µM NAD$^+$. Afterwards, bacterial replication was assessed by CFU assay. Statistics: two-tailed paired t-test. N = 3 biologically distinct samples (C,D), 4 biologically distinct samples (A, B, E); box plots: line at mean; box ranges from min to max; results are normalized against untreated controls.

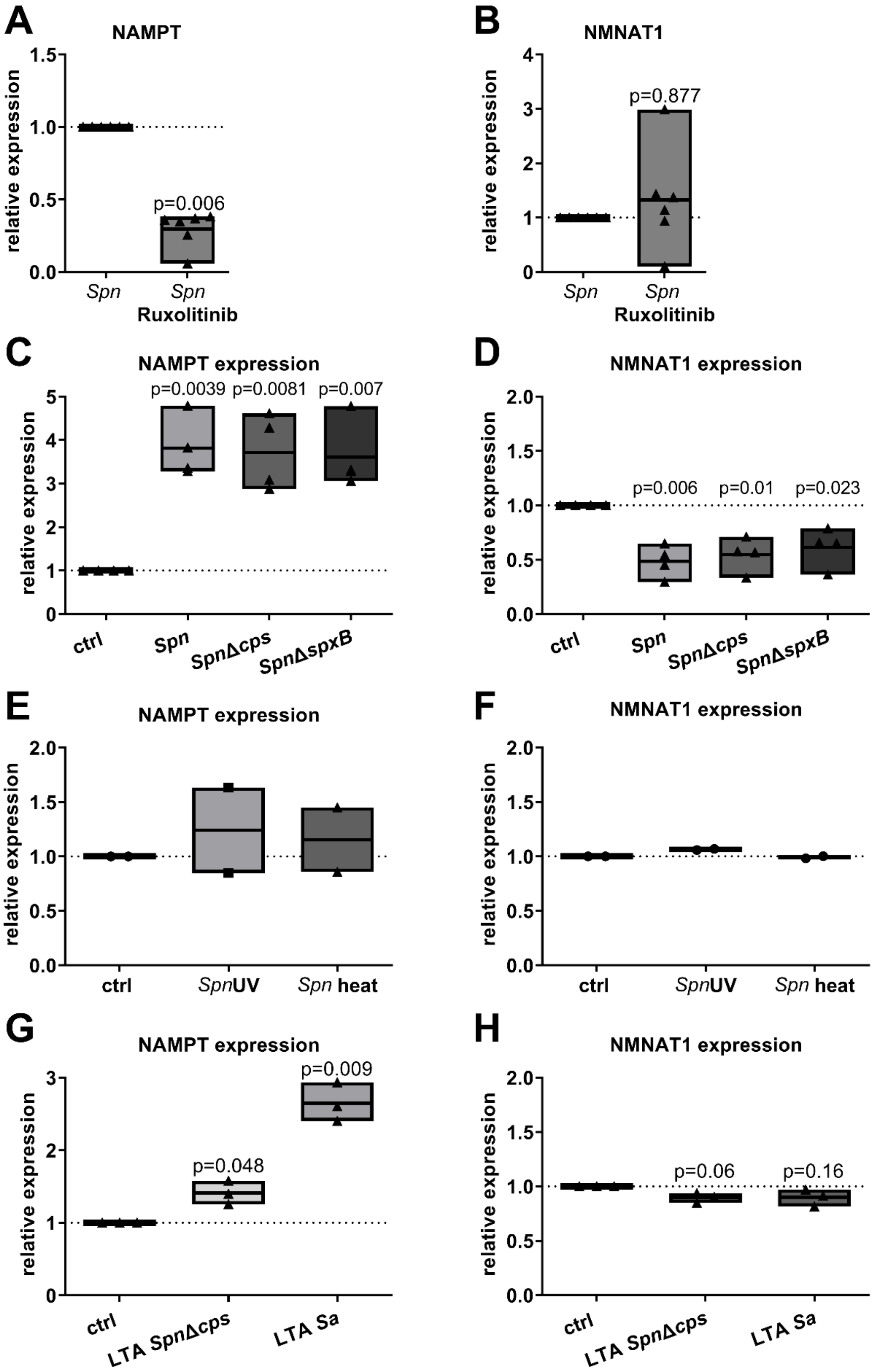

Supplementary Figure 5. NAMPT and NMNAT1 are regulated by distinct mechanisms. A, B) BEAS-2B were infected with Spn with or without Ruxolitinib (10 µM) treatment for 16 h. RNA was isolated and expression of

NAMPT and NMNAT1 determined. C, D) BEAS-2B were infected with indicated strains of Spn, D39 for 16 h. RNA was isolated and the expression of NAMPT and NMNAT1 determined. E, F) BEAS-2B cells were treated with heat- or UV-killed Spn D39 as indicated for 16 h. RNA was isolated and the expression of NAMPT and NMNAT1 determined. G, H) BEAS-2B cells were treated with LTA isolated from Spn D39 or S. aureus 113 for 16 h. RNA was isolated and the expression of NAMPT and NMNAT1 determined. Results are normalized to the house keeping gene RPS18 and depicted relative to untreated control cells. Statistics: two-tailed paired t-test (A, B), One-way ANOVA with Fisher's LSD (C-H); Significances were determined against untreated controls if not indicated otherwise. N = 2 biologically distinct samples (E, F); 3 biologically distinct samples (G, H); 4 distinct samples (C, D); 6 biologically distinct samples (A, B); box plots: line at mean; box ranges from min to max; results are normalized against untreated controls.

Source Data for supplementary figure 2

Western Blots:

42 h

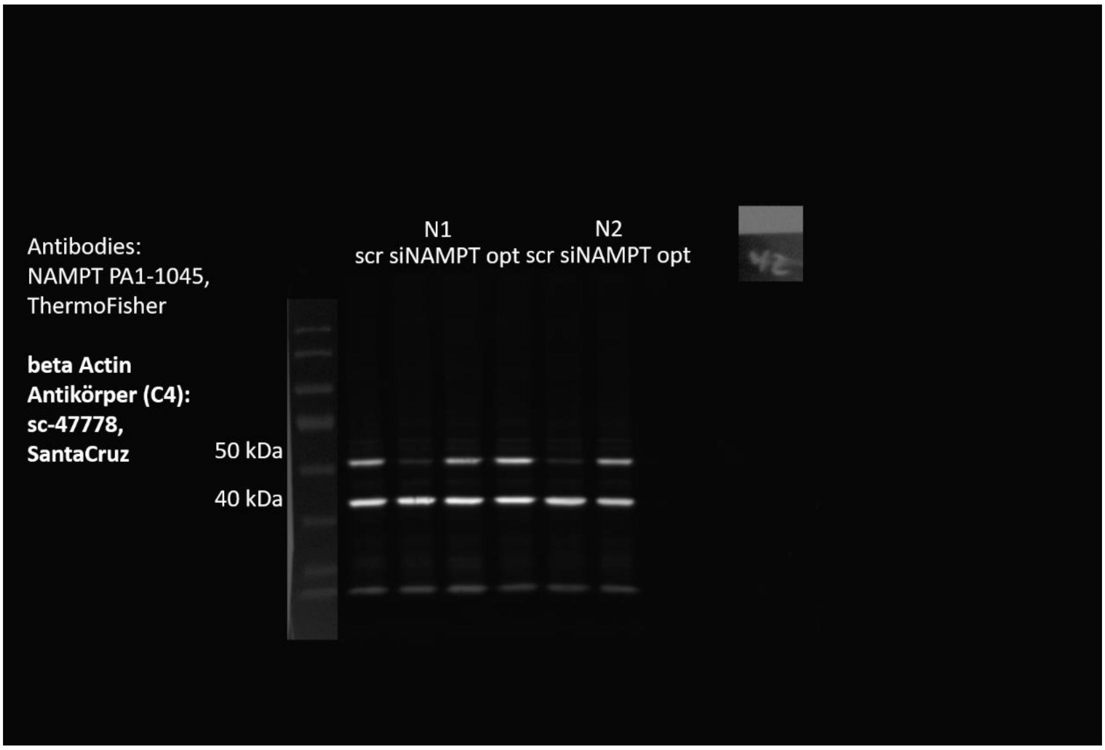

66 h

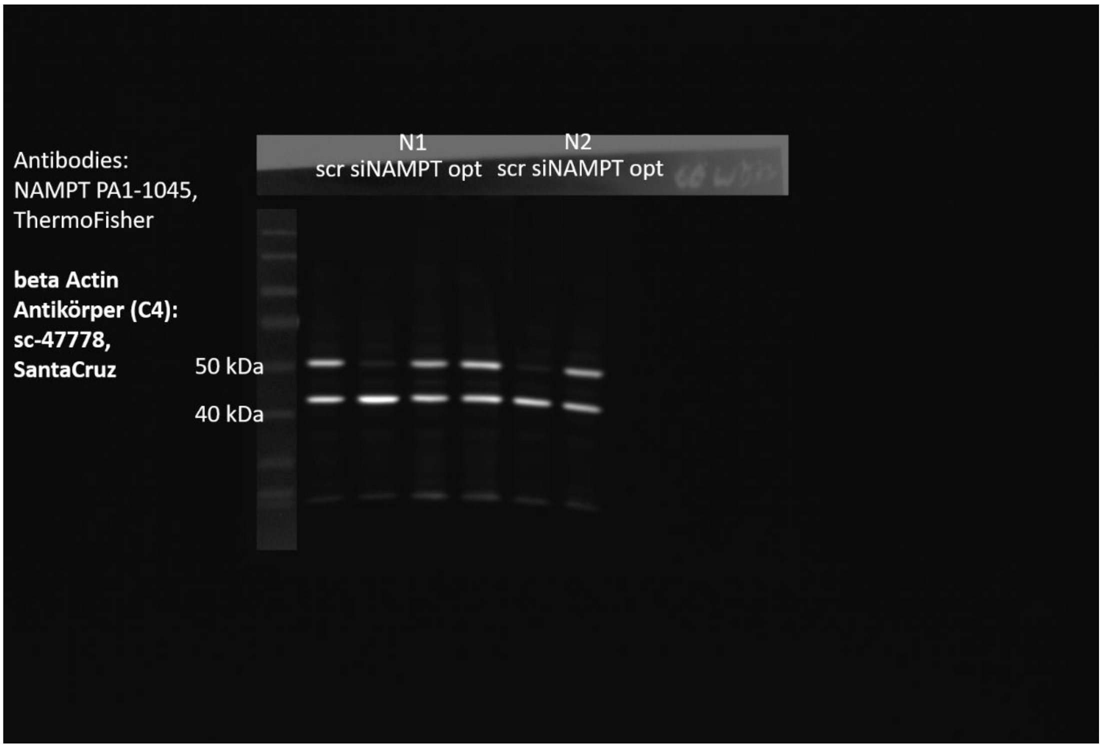

Supplement: Supplementary file 1 — Supplementary Information [file 41467_2023_41372_MOESM1_ESM.pdf]
