# Supplementary material for: NAD+ metabolism is a key modulator of bacterial respiratory epithelial infections
Source: Nat Commun. 2023 Oct 2;14:5818. doi: 10.1038/s41467-023-41372-w (PMC10545792; doi:10.1038/s41467-023-41372-w)
Supplement: Supplementary file 3 — Reporting Summary [file 41467_2023_41372_MOESM3_ESM.pdf]

nature portfolio

Corresponding author(s): Bernd Schmeck, Birke Benedikter

Last updated by author(s): Aug 10, 2023

# Reporting Summary

Nature Portfolio wishes to improve the reproducibility of the work that we publish. This form provides structure for consistency and transparency in reporting. For further information on Nature Portfolio policies, see our Editorial Policies and the Editorial Policy Checklist.

## Statistics

For all statistical analyses, confirm that the following items are present in the figure legend, table legend, main text, or Methods section.

| n/a | Confirmed | |
|---|---|---|
| ☐ | ☒ | The exact sample size (*n*) for each experimental group/condition, given as a discrete number and unit of measurement |
| ☐ | ☒ | A statement on whether measurements were taken from distinct samples or whether the same sample was measured repeatedly |
| ☐ | ☒ | The statistical test(s) used AND whether they are one- or two-sided<br>*Only common tests should be described solely by name; describe more complex techniques in the Methods section.* |
| ☐ | ☒ | A description of all covariates tested |
| ☐ | ☒ | A description of any assumptions or corrections, such as tests of normality and adjustment for multiple comparisons |
| ☐ | ☒ | A full description of the statistical parameters including central tendency (e.g. means) or other basic estimates (e.g. regression coefficient) AND variation (e.g. standard deviation) or associated estimates of uncertainty (e.g. confidence intervals) |
| ☐ | ☒ | For null hypothesis testing, the test statistic (e.g. *F*, *t*, *r*) with confidence intervals, effect sizes, degrees of freedom and *P* value noted<br>*Give P values as exact values whenever suitable.* |
| ☒ | ☐ | For Bayesian analysis, information on the choice of priors and Markov chain Monte Carlo settings |
| ☒ | ☐ | For hierarchical and complex designs, identification of the appropriate level for tests and full reporting of outcomes |
| ☒ | ☐ | Estimates of effect sizes (e.g. Cohen's *d*, Pearson's *r*), indicating how they were calculated |

*Our web collection on statistics for biologists contains articles on many of the points above.*

## Software and code

Policy information about availability of computer code

| | |
|---|---|
| Data collection | Mapix (Innopsys, 6.5.0), QuantStudio Real-Time PCR Software (ThermoFisher, v1.3); Magellan (Tecan, v7.1) |
| Data analysis | Basic Variant Detection tool (Qiagen, v2.2); CLC Genomic Workbench (Qiagen, v21.0.3); Proteome Discoverer™ (Thermo Fisher Scientific v1.4); MassHunter (Agilent, v10.0); GraphPad Prism (GraphPad Software, v9.5); R (v4.3) with limma package; Microsoft Excel 2016 |

For manuscripts utilizing custom algorithms or software that are central to the research but not yet described in published literature, software must be made available to editors and reviewers. We strongly encourage code deposition in a community repository (e.g. GitHub). See the Nature Portfolio guidelines for submitting code & software for further information.

## Data

Policy information about availability of data

All manuscripts must include a data availability statement. This statement should provide the following information, where applicable:

- Accession codes, unique identifiers, or web links for publicly available datasets
- A description of any restrictions on data availability
- For clinical datasets or third party data, please ensure that the statement adheres to our policy

The mRNA microarray data generated in this study have been deposited in the GEO accession viewer under accession code GSE195778 [https://www.ncbi.nlm.nih.gov/geo/query/acc.cgi?acc=GSE195778]. The proteome data generated in this study have been deposited in the PRIDE database under the accession number PXD039059 [https://www.ebi.ac.uk/pride/archive/projects/PXD039059]. Bacterial sequencing data generated in this study have been deposited

in the BioStudies database under the accession number E-MTAB-12644 [https://www.ebi.ac.uk/biostudies/arrayexpress/studies/E-MTAB-12644?key=b32e2efd-ee57-454b-afb9-33738809f574]. Source data are provided with this manuscript. Biological material and bacterial strains are available from Bernd Schmeck upon reasonable request.

## Research involving human participants, their data, or biological material

Policy information about studies with human participants or human data. See also policy information about sex, gender (identity/presentation), and sexual orientation and race, ethnicity and racism.

| | |
|---|---|
| Reporting on sex and gender | Conclusions in this study were drawn from data obtained by a) an immortalised cell line of male origin; b) previously published mouse data, obtained from sex matched or female only mice; c) primary lung cells/tissue, obtained from donors of male and female sex, according to self-report. As our results were reproducible over all models tested and the NAD+ salvage pathway is highly conserved accross all eukaryotes, we conclude that the results apply sex-independent. No sex-segregated analyses were performed due to local patient data protection laws and insufficient sample size. Information on the patients gender was not collected. |
| Reporting on race, ethnicity, or other socially relevant groupings | The manuscript does not use any variables drawn from race, ethnicity or other socially relevant groupings. |
| Population characteristics | Material was obtained from patients of the lung surgery unit, Marburg and Giessen University Hospital. As such, donors are between 60-80 years old, of male and female sex according to self-report, predominantly of caucasian descent and were previously in treatment for various lung-related morbidities. |
| Recruitment | Within the timeframes for recruitment, all patients were asked for their willingness to participate in the study. Therefore, we do not assume any specific recruitment bias. |
| Ethics oversight | Donated tissue was handled in accordance to local ethics regulations (Philipps University Marburg; permit number: AZ 224/12 for work with primary human bronchial epithelial cells; permit number AZ 161/17 for work with lung explants) and analysed anonymously. |

Note that full information on the approval of the study protocol must also be provided in the manuscript.

# Field-specific reporting

Please select the one below that is the best fit for your research. If you are not sure, read the appropriate sections before making your selection.

☒ Life sciences ☐ Behavioural & social sciences ☐ Ecological, evolutionary & environmental sciences

For a reference copy of the document with all sections, see nature.com/documents/nr-reporting-summary-flat.pdf

# Life sciences study design

All studies must disclose on these points even when the disclosure is negative.

| | |
|---|---|
| Sample size | No statistical calculation was performed to determine sample size. Instead, the number of replicates was chosen based on previously published studies (DOI: 10.1183/09031936.00186911 ; https://doi.org/10.1093/infdis/jiu806 ), our experience with the infection experiments and the observed effect strength. In general, at least 3 replicates were performed. At least 4 replicates using material from 4 different donors were performed when primary epithelial tissue was used for experiments. Additional N were performed in case of small effect sizes or high variation between replicates. 6 N were performed for Metabolome analysis, as the metabolites in question can be highly volatile (https://doi.org/10.1021/acs.analchem.9b00217 ). |
| Data exclusions | Replicates were excluded if they did not match quality criteria (RNA quality, proinflammatory marker expression). |
| Replication | Data were replicated at least 3 times with various passages of immortalised cell lines as well as donated tissues obtained from various donors. Gene regulations in BEAS-2B cells were further repilicated with two different cell batches, obtained from different sources. All attempts at data replication were successfull. |
| Randomization | In the study, experiments were done pair-wise with infected/treated cells/tissue being directly compared to untreated cells/tissue of the same passage/donor. Therefore, no distribution in study groups was performed |
| Blinding | As the study required direct infection of eukaryotic tissue by the investigator, no blinding was performed during the infection experiments. Investigator blinding was performed for analysis of metabolome MS data and electron microscopy. |

# Reporting for specific materials, systems and methods

We require information from authors about some types of materials, experimental systems and methods used in many studies. Here, indicate whether each material, system or method listed is relevant to your study. If you are not sure if a list item applies to your research, read the appropriate section before selecting a response.

## Materials & experimental systems

| n/a | Involved in the study | |
|---|---|---|
| ☐ | ☒ | Antibodies |
| ☐ | ☒ | Eukaryotic cell lines |
| ☒ | ☐ | Palaeontology and archaeology |
| ☒ | ☐ | Animals and other organisms |
| ☒ | ☐ | Clinical data |
| ☒ | ☐ | Dual use research of concern |
| ☒ | ☐ | Plants |

## Methods

| n/a | Involved in the study | |
|---|---|---|
| ☒ | ☐ | ChIP-seq |
| ☒ | ☐ | Flow cytometry |
| ☒ | ☐ | MRI-based neuroimaging |

# Antibodies

Antibodies used

PRIMARY: rabbit anti-NAMPT IgG, ThermoFisher, Cat.Nr. PA1-1045, LotNr. SG256353; goat anti-Actin, SantaCruz, Cat.Nr. sc-47778, various lots
SECONDARY: mouse Anti-rabbit IgG, HRP-linked, SantaCruz, Cat.Nr. sc-47778, various lots; donkey Anti-goat IgG, HRP-linked Santa Cruz, Cat.Nr. sc-2020, various lots

Validation

Anti-Nampt:
According to manufacturer's website: PA1-1045 detects Visfatin (PBEF) in human samples. PA1-1045 has been successfully used in Western blot procedures. In Western blot analysis of human adipose tissue lysate this antibody detects a ~52 kDa protein representing Visfatin. (https://www.thermofisher.com/antibody/product/NAMPT-Antibody-Polyclonal/PA1-1045, last accessed 29.06.2023)
Previously used in study: doi: 10.3390/ijms22136719

Anti-Actin
From manufacturer's website: -Actin Antibody (C4) is an IgG1 κ mouse monoclonal beta-Actin antibody that detects the beta-Actin protein of mouse, rat, human, avian, bovine, canine, porcine, rabbit, Dictyostelium discoideum and Physarum polycephalum origin by WB, IP, IF, IHC(P) and ELISA. β-Actin is highly conserved among species, making the β-Actin Antibody (C4) suitable for detection of β-Actin expressed in both mammalian and non-mammalian cells. (https://www.scbt.com/de/p/beta-actin-antibody-c4, last accessed 09.08.2023)

According to manufacturer used in more than 13.000 scientific publications; list available here: https://www.scbt.com/de/p/beta-actin-antibody-c4

# Eukaryotic cell lines

Policy information about cell lines and Sex and Gender in Research

| | |
|---|---|
| Cell line source(s) | BEAS-2B: obtained from ATCC (CRL-9609) and Sigma Aldrich (95102433) |
| Authentication | Cell lines were not further authenticated. |
| Mycoplasma contamination | Cells tested negative for Mycoplasma contamination. |
| Commonly misidentified lines (See ICLAC register) | No commonly misidentified cell lines were used. |
